# Prevention of krait bites by sleeping above ground: preliminary results from an observational pilot study

**DOI:** 10.1186/s12995-017-0156-7

**Published:** 2017-03-27

**Authors:** Chaturaka Rodrigo, Selvanayagam Kirushanthan, Ariaranee Gnanathasan

**Affiliations:** 1 Department of Clinical Medicine, Faculty of Medicine, University of Colombo, 25, Kynsey Road, Colombo 08, Sri Lanka; 2 Jaffna Teaching Hospital, Jaffna, Sri Lanka

**Keywords:** Krait bites, Sri Lanka, Poverty, Prevention, Snakebite

## Abstract

**Introduction:**

Neurotoxic envenoming following the bites of kraits (*Bungarus* spp.) is a common cause of death in the dry zone of Sri Lanka and elsewhere in South Asia. Most of these bites occur at night and are inflicted on people sleeping on the ground. Thus we hypothesized that the simple measure of sleeping above ground would help to reduce the number of observed krait bites.

**Methods:**

This study was conducted in two villages of the Kilinochchi district of Sri Lanka which had reported a high number of krait bites in the two years preceding the study. Most of the residents in these two villages slept on the ground. Residents in one area were given beds free of charge, using funds available from the study. Both villages received health education on the prevention of krait bites.

**Results:**

Forty five beds were distributed to 45 families in one village. This enabled 115 individuals to sleep above ground level. 6 monthly follow up visits were conducted ensuring the proper utilization of beds. Follow up was continued for 30 months (September 2013–March 2016); during this time period no krait bites were reported in either area.

**Conclusions:**

We observed a dramatic decline of krait bites in both villages. Better awareness with effective health education and clearing of vegetation could have led to the decline in the number of krait bites in both villages.

## Introduction

Over 400,000 envenoming events are estimated to occur globally, per annum due to snakebites [1]. Of the total envenoming events, more than 100,000 occur in South and Southeast Asia [1]. In Sri Lanka, an estimated 398 (95% confidence interval: 356–441) snakebites per 100,000 population occur annually and approximately 38% of them result in envenoming [2]. The estimated deaths due to snakebite in Sri Lanka are 2.3 (95% CI: 0.2–4.4) per 100,000 population [2]. Most of these envenoming events are due to cobra, Russell’s viper, saw scaled viper and krait bites. Regarding the geographical distribution of snakebite, the three provinces in the dry zone of Sri Lanka; North Central, Northern and Eastern provinces record the highest number of envenoming snakebites, respectively [2].

Snakebite envenoming caused by kraits (*Bangarus* spp.) is a major cause of morbidity and mortality in the dry zone of Sri Lanka, and elsewhere in South and Southeast Asia [3–6]. There are two species of krait in Sri Lanka; the Sri Lankan krait (*Bungarus ceylonicus*, endemic to Sri Lanka) and the common krait (*Bungarus caeruleus*) [5]. Both have neurotoxic venom that can cause death by respiratory paralysis [7, 8]. The onset of neuromuscular paralysis is rapid and can last for days while a subclinical neurological dysfunction can last for weeks [9]. A prospective study carried out by Ariaratnam et al. [5] studied 88 common krait bites in Sri Lanka and reported features of neurotoxic envenoming in 84 (95%) patients. In another case series in Sri Lanka, 75% of the krait bite victims developed neurological manifestations ranging from mild ptosis to respiratory paralysis [9]. An observational study in Anuradhapura district in the dry zone of Sri Lanka, reported 210 common krait bites in farmers over a period of three years. Respiratory paralysis was reported in 101 (48%) patients and 16 (7.8%) patients succumbed to the envenoming. Krait bites are usually painless and occur at night. In the previous study by Ariaratnam et al., all bites occurred indoors and at night (between 2300 and 0500 h) when the victims slept on the ground.

In the Northern province of Sri Lanka, which has the second highest risk of envenoming by snakebite in Sri Lanka, common krait bites are frequent. Sleeping on the ground on a mat is a common practice among the poor families in this area who cannot afford a bed. Especially in the districts of Kilinochchi, Mannar, Vavuniya and Mullaitivu, the residents had been displaced and lived as refugees for many years due to the armed conflict that lasted from 1981 to 2009. After the conflict had ended, residents returned to their previous houses which had been abandoned for years. Most of them did not have the financial resources to afford beds and were sleeping on the ground at night. Based on the observations by Ariaratnam et al. [5] we presumed that sleeping on the ground would increase their risk of envenoming by krait bites. The aim of this study was to observe if sleeping at least 30 cm above ground would lead to a reduction in the number of krait bites in a village in the Kilinochchi district of the Northern Province of Sri Lanka.

## Methods

Two villages from the Kilinochchi district of the Northern Province of Sri Lanka (one of the five districts the Province) were selected for the study. An analysis of snakebite cases from the local hospital over 6 months showed that rural Kilinochchi had a high incidence of snakebite. However hospital statistics were not detailed enough to identify the high incidence villages. Thus a survey was carried out among several randomly selected villages in Kilinochchi district within a 30 km radius of the Kilinochchi base hospital to identify two villages that were suitable for inclusion in the study (with frequent reports of krait bites and more than 90% of people sleeping on the floor). One of the investigators visited each area to assess suitability. The selected two villages collectively reported 21 snakebites over the preceding two years with 10 krait bites (data collected by a household survey). These numbers were self reported and could not be confirmed independently. One village was randomly assigned to receive beds (V1) while the other village (V2) was observed for comparison. V1 was KN 03 Kanakaipuram East house plan (9^0^38′ N 80^0^30′ E, elevation: 26 m) and V2 was KN 03 Kanakaipuram West house plan (9^0^40′ N 80^0^27′ E, elevation: 26 m). The two villages were approximately 3.5 km apart from each other.

The calculated sample size for V1 was 110 (assuming a krait bite prevalence of 2.5 per 100 per year). In phase one of the study, an assessment of demographics and sleeping habits of residents in both areas was completed. At the same time a health education programme on snakebite prevention was carried out for both V1 and V2. In this programme, one of the investigators addressed the entire village in a community gathering and explained about preventive measures against all snakebites. It was stressed that the surroundings should be kept clear of potential dwellings for snakes. A leaflet in Tamil (native language) was distributed among all families with key information on behavioural interventions to prevent snakebites (sleeping above ground level, wearing boots, avoiding bush walking at dusk or at night and treading heavily and carrying a stick if bushwalking cannot be avoided) as well as information on the identification of kraits and first aid in the event of a snakebite. The health education was repeated by the same investigator (to ensure consistency) during follow up visits. In phase two, the number of beds required for the V1 was calculated and the beds were distributed. The number of beds donated depended on (a) available funds, (b) spatial restrictions (some houses were so small that a large bed would not fit in) and (c) the maximum number of people who were able to sleep above ground using a single bed. The best combination of single and double beds was determined based on the assumption that a double bed would accommodate a mother and two small children while a single bed would accommodate a mother and a child. The beds were distributed among all families in V1 (one bed per family to ensure an equal distribution as limited funding prevented giving beds to all residents).

Follow up was conducted at 6 monthly intervals to ensure proper utilization of beds. Health education (an informal discussion at family level, reiterating the points raised at the initial educational session) was also carried out during follow up. V2 was also visited at the same time to record the number of snakebites as well as for periodic health education. The beds were distributed in September 2013 and six-monthly follow-up visits and health education programmes were carried out until March 2016 with a total follow up of 30 months.

## Results

**Fig. 1:**
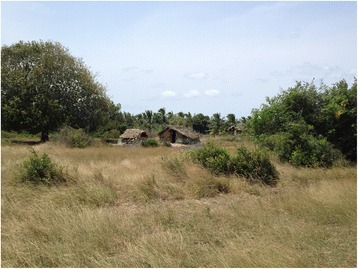
Photograph of a typical house in the village that received beds

V1 had 46 families (214 individuals) and V2 had 50 families (222 individuals). In V1, 21 families had more than 5 members and 29 families less than 5 members. There were 76 children under 12 years of age in the village. In both villages, only two families had a bed in their house (one each per village). Most houses in the villages were very basic dwellings made from mud walls and cadjan thatched or tin roofing (and a few small, single room, brick walled houses). Most houses could not accommodate more than one bed. The houses were located away from each other and on most occasions were separated by a thick growth of vegetation (Fig. 1). The original forest cover in these areas is classified as dry monsoon forests but this had been largely destroyed due to fires and destruction during the armed conflict. The two nearest hospitals; Akkarayankulam divisional hospital and Kilinochchi district general hospital were 10 and 23 km away from V1, respectively.

**Fig. 2:**
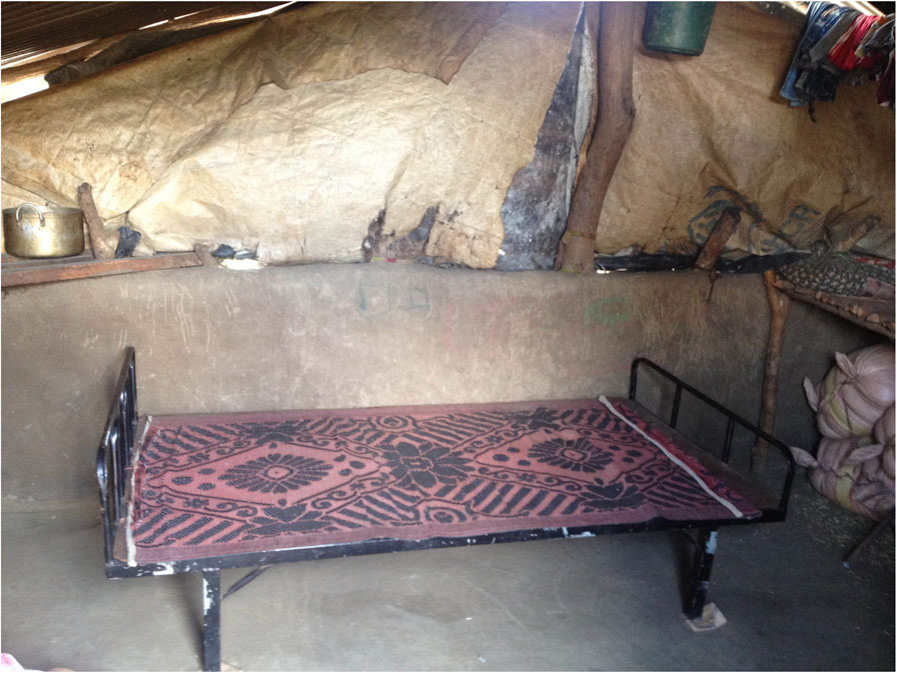
Photograph of a donated bed within a house (the positioning of the bed against the wall is not ideal as snakes may crawl using surrounding structures as support. However, spatial constraints within some houses prevented proper positioning of beds)

Forty five beds were distributed to 45 families (one bed per family, Fig. 2). The best combination of beds (to maximize the number of people sleeping above ground) was decided based on the family structure. Twenty five double beds and 20 single beds were distributed in the expectation that at least 115 individuals would sleep above ground.

**Fig. 3:**
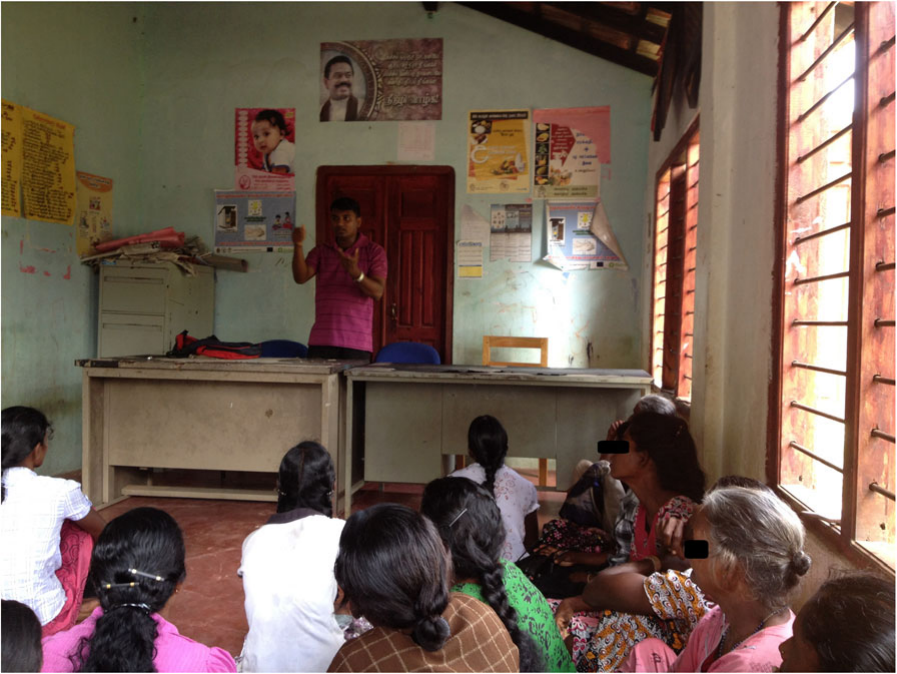
A session of health education and community awareness during the project

The health education programme was well received and at least one member from all families participated (Fig. 3). All families in V1 were observed to utilize the beds appropriately during follow up. While the investigators suggested how bed use could be optimized, the residents were free to use it according to their needs. In all homes children and mothers received priority. Interestingly, over the 30 month observation period no krait bites was reported from either V1 or V2. There had been two snakebites, one each from V1 and V2, but on both occasions the snake was not identified and there was no evidence of envenoming.

## Discussion

This purpose of this study was to observe if sleeping above ground level would reduce the incidence of krait bites in a village located in Kilinochchi district of the Northern Province in Sri Lanka. Previous observations had shown that krait bites occurred at night and when the victim was sleeping on the floor. Due to poverty, people living in these villages could not afford beds. In this study, beds were donated to one village while another nearby village (presumably at the same risk of snakebite) was observed. Health education on avoiding snakebite was offered to both villages. At the end of a 30 month observation, both villages did not report any krait bites.

This study targeted a relatively low cost behavioral intervention which would also improve the quality of life of participants. It did not require any extensive environmental modifications but only a behavioural adjustment on the part of the participants. The value of such behavioural modifications and health education in preventing snakebites has been shown previously in South Asia. In Nepal, a survey of 11,176 households that assessed living conditions and its correlation with likelihood of snakebites, concluded that sleeping under a bednet reduced the likelihood of a snakebite significantly (OR: 0.02; 95% CI: 0.007–0.07, *p* < 0.0001) [10]. In another non randomized before and after study in rural Nepal, the impact of health education and rapid transport of victims to a healthcare center by motorcycles (by volunteers) was assessed [11]. Mortality rates were reduced with intervention due to timely medical care as expected but surprisingly, the incidence of snakebites also showed a significant reduction. Even though fast transport of victims can explain the improved mortality rates, it cannot explain the reduced incidence of snakebites, which may be due to effective health education [11]. Wearing enclosing shoes or boots is another seemingly intuitive method to prevent snakebite. However, compliance with footwear is much more difficult to assess and requires close follow up. A systematic review that assessed the impact of footwear on preventing snakebite could not come to a conclusion due to inadequate data [12].

With this background, and after considering the observations of krait bite epidemiology in Sri Lanka, we concluded that bed donation would be an effective strategy that can be reliably monitored over time. With the available funding, we managed to donate enough beds to enable an adequate sample size to sleep above ground level. Despite previous studies showing health education to significantly reduce incidence of snakebite (hence a potential confounder in observations), we opted to give health education to both villages due to ethical reasons. During the 30 month follow up, there was a dramatic reduction in snakebites. However, given that the same reduction was observed in the control village it is not possible to attribute this to the bed donation alone. However, it is highly likely that the change in sleeping behaviour would have contributed to the observed reduction in snakebite.

There are two other reasons that might have contributed to the observed results (apart from the effect of health education). Clearing of vegetation around houses was visible in both villages as people were returning to their homes after the war and this would have resulted in a habitat reduction for kraits. The population in the Kilinochchi district was approximately 197,000 in 2006 but dropped to 23,000 due to a massive displacement in 2009 when fighting involved the district in the last stages of the war [13]. The population of the district had recovered to approximately 113,500 by the time the first post-war census was conducted in 2011/2012 [14]. This had increased further to 122,000 by 2016 (estimated mid-year population) when this study concluded [15]. Secondly, the possibility of an underpowered sample is also likely as our initial assessment of krait bite incidence was dependent on self reported data from the two villages. However, the residents of these areas are very familiar with common kraits. Also, krait bites have a distinctive epidemiological pattern and a clinical symptomatology that is difficult to miss.

Overall, we believe that a larger study avoiding the limitations of this study (i.e. larger sample size, better estimate of background snakebite incidence and selecting cases and controls from the same geographic and climate zones but further apart from each other) would be helpful in getting a reliable estimate of the impact of sleeping above ground for krait bite prevention.

## Conclusion

This prospective observational study donated beds to one village located in the Killinochchi district of the Northern Province of Sri Lanka with the objective of reducing krait bites by promoting sleeping above ground level. A nearby second village was observed without bed donation but both villages received similar health education on snakebite prevention. After a follow up of 30 months, a dramatic reduction of snakebites was observed in both villages. Since this observation was common to both villages, the reduced incidence of bites cannot be attributed to bed donation alone, although it was highly likely to be a contributing factor. The value and effectiveness of this innovative low cost intervention needs further exploration in a larger study.
